# Growing up in a Rare Disease Family: The Perspectives of Adolescent and Young Adult Siblings

**DOI:** 10.1002/jgc4.70328

**Published:** 2026-11-13

**Authors:** Melissa Goosen, Shahida Moosa, Chrisma Pretorius

**Affiliations:** ^1^ Department of Psychology Stellenbosch University Stellenbosch South Africa; ^2^ Faculty of Medicine and Health Sciences, Division of Molecular Biology and Medical Genetics, Tygerberg Hospital Stellenbosch University Cape Town South Africa

**Keywords:** adolescent, experience, rare disease, siblings, young adult

## Abstract

The implications of a rare disease (RD) extend beyond the individual who has been diagnosed. Siblings face many challenges associated with having a brother or sister with an RD, yet our understanding of their experiences is limited, especially within the South African context. This qualitative study aimed to explore the experiences of adolescent and young adult siblings of individuals with RDs in Cape Town, South Africa. The study focused on the challenges siblings experience, explored how siblings navigate these challenges, and examined how family organization, such as communication processes, influence their experiences. Purposive sampling was used to recruit eight siblings of individuals with RDs at Tygerberg Hospital, Cape Town. Semi‐structured interviews were conducted with these participants, aged 11–19 years, and the data were analyzed using reflexive thematic analysis (RTA). The Revised Theory of Creating a Tenuous Ba was used as a theoretical point of departure. The following four themes were generated, as they were central to the experience of having a sibling with an RD: (1) becoming an adjunct caregiver; (2) making sense of RDs, (3) negotiating relationships shaped by an RD; and (4) managing the emotional impact of an RD. The findings suggest that having a brother or sister with an RD has implications for the emotional and social wellbeing of siblings. Siblings act as adjunct caregivers and should, thus, be considered integral to the caregiving unit. Due to a shortage of age‐appropriate information and limited communication in RD families, siblings lack sufficient support from parents and peers to navigate their complex experiences. The findings assist in building a foundation for intervention development that targets siblings and RD families in South Africa.

## Abstract (highlights)

**What is Known About This Topic?:**

Current international research on the impact of a rare disease (RD) focuses on the individuals diagnosed and their caregiving unit (the family members and other individuals who provide day‐to‐day care). Siblings face obstacles associated with having a brother or sister with an RD that negatively impact their emotional and social wellbeing, yet they are often overlooked in research and frequently excluded from information provision and support interventions. Although some studies have been conducted to explore the experiences of siblings in high‐income countries, there is a paucity of research in low‐ and middle‐income countries such as South Africa.

**What this Paper Adds to the Topic?:**

This qualitative study provides first‐hand accounts of the experiences of South African siblings of individuals with RDs. This study explores how siblings experience the challenges posed by having a brother or sister diagnosed with an RD, provides an understanding of how siblings navigate these challenges, and investigates how family organization, such as communication processes, influence their experience. The findings demonstrate that siblings should be considered as integral to the caregiving unit and assist in building a foundation for much‐needed intervention and support.

**Abbreviations**
RD: rare disease
RTA: reflexive thematic analysis

## 1 Introduction

Rare disease (RD) is a collective term used to describe a heterogeneous set of conditions which respectively affect a small number of individuals (Schieppati et al. 2008). In order to be classified as an RD, the European Organization for Rare Diseases, a governmental alliance of patient organizations in Europe, requires a prevalence rate of less than 1 in 2000 citizens per country (European Organization for Rare Diseases 2005). Although RDs individually affect a small number of people, their collective impact is much greater (Ferreira 2019). With over 7000 recognized RDs, an estimated 4.2 million South Africans suffer from an RD or will be afflicted with one during their lifetime (Malherbe 2023; Moosa 2022; Moosa, personal communication, February 22, 2024).

The impact that RDs have on the family system, especially siblings, is a theme that has recurred in recent research (Haukeland et al. 2021). Current international research on the impact of an RD focuses on the individuals diagnosed and their caregiving unit (Heaton et al. 2023; Hutson and Alter 2007). Siblings face obstacles associated with having a brother or sister with an RD which have been found to negatively impact their emotional and social wellbeing (Haukeland et al. 2015, 2021), yet they are often overlooked in research and are frequently excluded from information provision or support interventions (Hutson and Alter 2007). The experiences of adolescent and young adult siblings are of particular interest, as these are periods of dynamic identity exploration and formation of social attachments (Jaworska and MacQueen 2015; Settersten and Ray 2010), which may be impacted by the experience of having a sibling with an RD.

Although studies have been conducted to explore the experiences of siblings in high‐income countries (Haukeland et al. 2015; Heaton et al. 2023), there is a paucity of research in low‐ and middle‐income countries. South Africans face unique socioeconomic and psychosocial challenges that could exacerbate the challenges of an RD diagnosis (Jacobson 2022). A lack of RD specialists in rural areas (Jacobson 2022) and a shortage of local support groups for families could result in limited educational and emotional support for siblings. It is, therefore, crucial to include siblings in research on RD to allow for first‐hand accounts of their experiences, especially in the South African context. In this way, challenges that siblings face can be identified, allowing for future development of interventions. Accordingly, this study aimed to investigate the experiences of adolescents and young adults who have a sibling with an RD in Cape Town, South Africa with a focus on the following objectives:

1. Exploring the challenges posed by having a sibling diagnosed with an RD.
2. Understanding how siblings navigate challenges associated with having a sibling with an RD.
3. Investigating how family organization, such as communication processes, influences the experience of siblings.

## 2 Methods

The exploratory design of this study involved semi‐structured interviews with eight adolescent and young adult siblings of individuals with RDs at Tygerberg Hospital in the Western Cape, South Africa. Following the concept of information power by Malterud et al. (2016), the final sample size was considered sufficient to support meaningful interpretation of the siblings' experiences. The specificity of the sample, use of an established theoretical framework, and strong quality of dialogue from participants contributed to the strength of information power (Malterud et al. 2016). This study was approved by the Human Research Ethics Committee of Stellenbosch University (S24/01/015), and permission was obtained from Tygerberg Hospital to conduct the study on their premises.

### 2.1 Setting and Participants

Participants were recruited using purposive sampling. The professional networks of the authors at Tygerberg Hospital's Medical Genetics clinics in Cape Town, South Africa were used for recruitment. A research nurse working at Tygerberg Hospital distributed informational flyers to potential participants. The flyers informed participants about the aims of the study, necessary inclusion criteria, what participation would entail, and contact information for the first author. Some parents requested for contact to be made by the first author and provided their contact information. Participants needed to meet several requirements to be included in this study (Table 1). The broadest definition of a sibling was used to include biological, adopted, foster, and stepsiblings. This broad definition is commonly applied in research so that valuable narratives are not excluded based on the criteria of biological relation (Grant et al. 2021; Vatne et al. 2015).

**TABLE 1:** Inclusion criteria.

Participant requirements	The participant has at least one sibling diagnosed with an RD
	The participant does not have an RD themselves
	The participant resides within the same household as the siblings who has been diagnosed with an RD for more days than not
	The participant is between the ages of 10 and 24 years old

Once contact had been made, either by the parents of participants or by the first author, a meeting was arranged to obtain informed consent and assent, fill in a biographical information form, and conduct the interviews. Written consent was obtained using an informed consent form for participants older than 18. If the participant was a minor, they provided written assent while their legal guardian provided written consent. There were two versions of the assent forms, one for participants aged 10–13 years and one for participants aged 14–17 years, to ensure that the participants fully understood the study.

Each participant chose to have their interview in‐person in a private room at Tygerberg Hospital or online via Zoom/Microsoft Teams. If the participant was under the age of 18, they could have their legal guardian present during the interview, but none opted for this. Two interviews were conducted in‐person at Tygerberg Hospital, and six were conducted online. Author MG conducted all the interviews, which ranged from 25 min to 34 min, with the average length being 30 min. With verbal permission from participants, all interviews were recorded for transcription and data analysis purposes. Each participant was assigned a pseudonym by the research team to ensure anonymity.

### 2.2 Materials

A semi‐structured interview guide was developed by author MG. There were two versions, one for participants aged 10–13 years and one for participants aged 14–24 years. These versions were created to ensure participants could discuss their experiences in a manner appropriate to their age and level of development. For example, “What advice would you give to other children/adolescents/young adults who have a sibling with an RD?” for participants aged 14–24 was adapted to “What would you say to help other children who have a brother or sister with an RD?” for participants aged 10–13.

### 2.3 Data Analysis

This study was situated within an interpretivist and phenomenological paradigm, which considers reality to be socially constructed and seeks to describe, understand and interpret participants' subjective experiences (Ash 2025). Since this study aimed to gain an in‐depth understanding of siblings' complex experiences, this approach was deemed suitable. Coding and analysis of the interview data followed Braun and Clarke's (2019) principles of reflexive thematic analysis (RTA). Additionally, the Revised Theory of Creating a Tenuous Balance (Havill et al. 2019) was used inductively to code participant interviews and deductively to relate study findings to this theory. This revised theory highlights how healthy siblings of an individual with chronic illness and rare diseases strive to understand the illness, adapt to family changes, manage strong emotions, and maintain stability in their life (Havill et al. 2019). This hybrid approach to thematic analysis balances inductive and deductive coding and has demonstrated rigor in qualitative research (Fereday and Muir‐Cochrane 2006).

Following Braun and Clarke's (2024) six‐phase approach to thematic analysis, the interview recordings were first transcribed manually by MG to ensure familiarity with the data. During this initial process, MG actively engaged with the data by making notes about any insights gained. Second, MG generated initial codes that were rich and inclusive and noted those that were relevant to the Revised Theory of Creating a Tenuous Balance. Third, codes were examined and developed into themes and subthemes that represented patterns of meaning within the dataset. Fourth, these initial themes were reassessed in consultation with CP, ensuring a coherent interpretation of participants' experiences. Fifth, themes were finalized, defined with a brief synopsis and assigned a concise, informative name. The sixth and final step is the clear, logical write‐up of findings which will be discussed under findings.

### 2.4 Reflexivity, Transparency and Methodological Integrity

Author MG maintained reflexive awareness of her assumptions and recognized her influence on interpretive decisions. Her academic background (genetics and psychology) informed her interest in exploring experiences of siblings of individuals with RDs and provided a foundation for engaging with this research topic. It should be noted that she had no personal experience of living with or caring for someone with an RD and had no relationship with participants prior to conducting this study. She had previous experience working with children and adolescents in her voluntary roles at care centres and academic development programs in South Africa.

Consistent with Braun and Clarke's (2024) approach to RTA, reflexivity was embedded through reflexive journaling and regular supervisory discussions. Before and after each interview, MG maintained a journal to document her expectations, emotional responses, and emerging interpretations. Ongoing discussions with author CP supported a process of critical reflection on how these perspectives may influence data collection and interpretation.

Author MG assumed primary responsibility for the analytic process, including the recruitment of participants, design of the interview guide, conducting of interviews, and leading the data analysis. Emerging codes and themes were discussed regularly with author CP, contributing to the refinement and conceptual development of the findings. Author SM, who is a clinical geneticist, provided substantial contextual expertise throughout the study. All authors reviewed and approved the final manuscript.

## 3 Findings

Demographic characteristics of the eight participants in this study are summarized below (Table 2). The eight siblings were recruited from seven different families, with participant numbers six and seven being biological brothers. Five participants were male and three were female. The majority of participants were aged between 10–14 and were classified as early adolescents (Blum et al. 2014). Ages ranged from 11 to 19 years old with a median age of 12.5 years old.

**TABLE 2:** Demographic characteristics of participants.

	*n* = 8
Age (years)	
11–13	5
14–19	3
Self‐reported gender	
Female	3
Male	5
Home language	
Afrikaans	4
English	3
Xhosa	1
Rare disease classifications	
Chromosomal microdeletion	2
Multiple congenital abnormalities	3
Rare skin disorder	1
Rare bone disorder	2

As part of each semi‐structured interview, a brief genealogy provided context for the family structure of participants. Following the interview technique from Heaton et al. (2023), information on how many siblings the participant had, birth order and whether any extended family lived with them was collected. This information contextualized the findings, as family structure could influence how practical support and caregiving responsibilities were distributed due to the availability of other family members. Six of the participants were the first‐born child. All participants reported that only one of their siblings had been diagnosed with an RD. Two of the participants reported no additional brothers or sisters, while the rest had three or more siblings. Seven of the eight participants lived with both biological parents. One of the participants shared a home with extended family, including grandparents and aunts. All siblings noted that they had family that lived close by.

Four key themes were created during data analysis, namely: (1) becoming an adjunct caregiver; (2) making sense of RDs; (3) negotiating relationships shaped by an RD; and (4) managing the emotional impact of an RD. These themes and their relevant subthemes are summarized in Figure 1.

**FIGURE 1:**
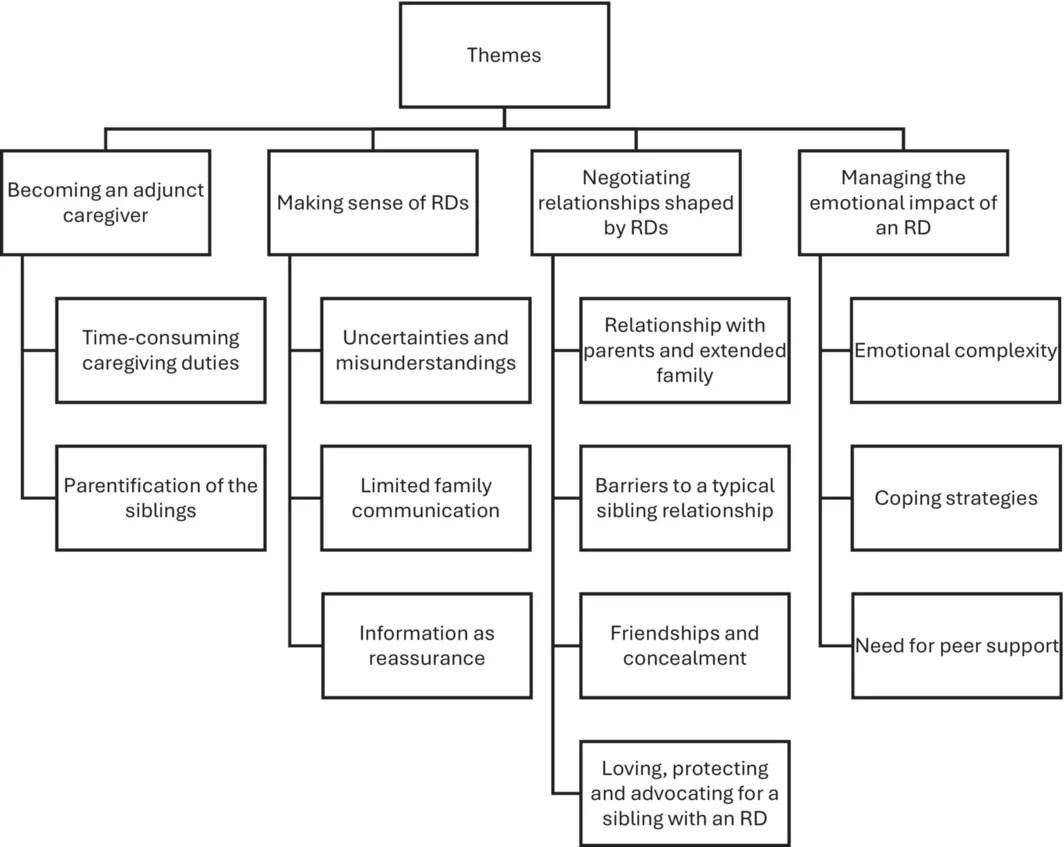
Visualization of themes.

### 3.1 Becoming an Adjunct Caregiver

Siblings gradually assumed caregiving responsibilities that extended beyond typical sibling roles, positioning them as adjunct caregivers within the family. Increased family responsibilities were related to household tasks and healthcare needs of their sibling diagnosed with an RD. Common activities included grocery shopping, assisting with hygiene practices such as bathing and the administration of medication. This theme is presented through two subthemes showing how siblings provide supplementary caregiving support: (1) time‐consuming caregiving duties; and (2) parentification of the sibling.

#### 3.1.1 Time‐Consuming Caregiving Duties

Siblings tended to take on caregiving duties that were previously carried out by their parents before the diagnosis of an RD. The parents of three participants had asked them to assist in these duties due to feelings of overwhelm and struggle to adapt to intensive healthcare needs:

Sometimes I offer and sometimes she [mom] tells me to do it. Max, 12M

I help because my mommy can't handle both of them [siblings]. Grant, 11M

Participants illustrated the demanding nature of these caregiving responsibilities, especially those that needed to be continuously executed:

I would say every day … it's like a routine. Every day the same thing … I clean his diaper, give him food. Jake, 14M

Every morning, he needs to drink his pill and then in the evening he needs to do a pump for his lungs and his nose spray. Jane, 19F

Given the need for constant caregiving for their sibling with an RD, participants described stepping in when their parents were at work or running errands. This often impacted their daily schedule, as caregiving tasks took precedence over other activities such as schoolwork or social activities:

When I worked or when I went to school, I did my homework after he was sorted out. Once he was dealt with then I could begin with other things … Then I would have time to do some of the things that I really wanted to do. Jane, 19F

If she [mom] goes to work, then I will always have the child [sibling] with me. If my dad is also at work, then there is nobody to look after him. I'm the only one. Ella, 12F

These accounts reveal how siblings tend to subordinate their own needs when taking care of their brother or sister with an RD and have limited time available to attend to their own interests and hobbies.

#### 3.1.2 Parentification of the Sibling

Siblings in this study often assumed a parent‐like role for their brother or sister with an RD. Parentification is a phenomenon in which children complete tasks that are typically reserved for adults, and participants reported performing tasks that can be classified as instrumental parentification (Hooper et al. 2011). Hooper et al. (2011) consider the following tasks inappropriate for the developmental age of the participants: bathing and helping their sibling with an RD use the bathroom, grocery shopping for the family, and assisting their sibling with their schoolwork:

I feel right because I must help them [siblings] also when my daddy is not there at home. I'm there with her [sibling with the rare disease] if she wants to go to the bathroom or get in the car and go to her cousin… When we need to go to the shop for them then I go get bread and if she wants something when she comes from school then I need to go for her. Grant, 11M

Then I say I can buy something for him… After school if my mom isn't there then I have maybe a R20 or R50, then I give it to my grandmother, and she will go buy something for us to eat. Ella, 12F

The role reversal described by siblings seems to be connected to the intimate bond that is formed between a sibling and their brother or sister with an RD:

I will do anything for him [sibling with RD]. Jake, 14M

For one participant, the sibling‐focused parentification extended to evaluating and contesting her mother's approach to care…Ella (12F) shared that she did not approve of the way her mother cared for her sibling with an RD:

Look, I really don't like it when my brother is sitting outside with my mom. I usually take him from her and go sit with him in our yard. When my grandmother comes to ask me why I have the child with me I tell her that my mom is sitting outside with her friends, and I don't like that.

Ella's account suggests that the conflict arose not from the caregiving tasks themselves but from differing views on what adequate care for her brother involved. By taking over his care, Ella positioned herself as his protector and implicitly took on a supervisory, parent‐like role in relation to her mother.

### 3.2 Making Sense of an RD

Siblings attempted to make sense of the way an RD diagnosis would affect their lives by relying on information and educational resources available to them. The three subthemes that illustrate this concept are (1) uncertainties and misunderstandings; (2) limited family communication; and (3) information as reassurance.

#### 3.2.1 Uncertainties and Misunderstandings

Siblings shared many uncertainties about their sibling's RD. They had unanswered questions, some that had been on their mind since before a diagnosis had been received. Faced with uncertainty, siblings seemed to form their own theories to explain the symptoms of their brother or sister:

I thought he had asthma or that something in him breaks easily… He is breakable, like his teeth are glass. He is made of glass. Ella, 12M

He had something in his lungs. Initially we thought that it had these effects on him. I just wondered, if it is something that you can get medication for then everything will be okay. It's bad for me because he has been a sickly child since birth, but we didn't expect this. We didn't expect what the actual problem was. Jane, 19F

Participants noticeably misunderstood the RD of their brother or sister. These misunderstandings ranged from the cause of the RD to how specific symptoms are manifested:

I am scared that I am also going to become like him. That we won't be able to understand each other or speak to each other… I would say that he has autism, so that you can just understand that he doesn't speak very well. Max, 12M

Then I will just tell you that I don't know what's going on with him. My mom didn't tell me. Ella, 12F

It is important to note that the parents and/or legal guardians of the participants were also uncertain about the RD. Although filling out the biographical information forms, parents did not know the name of their child's RD but could relay what doctors had explained to them. Misconceptions held by siblings could be due to inaccurate or incomplete explanations from parents. For some siblings, these misunderstandings led to feelings of distress and self‐blame surrounding their siblings' symptoms:

Maybe when I do something bad to him I will think… I thought that the disease will wake up and just sometimes he will not feel better. So I always do good for him. Ethan, 12M

#### 3.2.2 Limited Family Communication

Family communication was a salient aspect of sibling experiences as parents served as the primary conduits for information about RDs. All except one participant in this study reported that their parents were the first to tell them about the RD diagnosis:

My mother and father, I think they arranged a meeting to tell me and my older brother that he was born with this disease. I think, well they did not tell me full details, but they told me that he wasn't born like us. Ethan, 12M

I think I was still at school, and they were in Cape Town… She then told me there is a problem with him. That is why he is slow and everything. The told me that and I thought, oh my word, this really is something. Jane, 19F

After this initial discussion, there was infrequent family communication about their brother or sister's RD:

We don't really speak about it, nobody speaks about it at all actually. Layla, 18F

My mom is very strict with me. She is just going to tell me that I am still too young to talk about it… but when I am older, I am going to ask my mom about it. Ella, 12F

Jane was the only participant who spoke more about her sibling's RD as time went on. She reflected on how her family was originally ashamed to speak about her sibling's RD and how they had become more comfortable with it over the years:

I like to speak about it, about his disease. It's not that we are ashamed about it now. We were in the beginning but then we started to accept it. So now it is okay for me to talk to other people about it. 19F

#### 3.2.3 Information as Reassurance

Siblings highlighted that receiving more information about their brother or sister's RD would provide them with a sense of relief and peace of mind:

I feel good when I know what is happening with my brother… so that's very important to me. Max, 12M

The misunderstandings and infrequent family communication discussed in this theme did not lead to information seeking behavior for most participants in this study. Only two participants described doing their own research on RDs to aid in their understanding:

I did my own research. Nobody knows, not even I knew that humans had chromosomes until I got to high school and started taking biology. Then I understood his illness a lot better because I had biology. So, then I went and did some research about what [The name of the rare disease is a potential identifying factor and has been removed from the quote to ensure anonymity: rare disease name] is. Jane, 19F

At first, I think I was more confused. My mommy said it's because of the age she was that she had my sister. I didn't know it had a name… I did research when my mommy first told me. Layla, 18F

However, siblings noted that they would appreciate explanations from medical professionals about the implications of an RD:

In full detail since they are like the medical professionals… My mommy will like try to dumb it down… If the doctors explain it to me, they would use like medical terms and all that stuff Because maybe with the full details then I know if there is complications, what the complication would be. Layla, 18F

I think I would love to have them explain for me because they know it better than my parents. They can explain it better because I think it's them who found the disease in my brother so then they know about how the doctor works and how the body of a child works. Ethan, 12M

These quotes illustrate a need for siblings to be included in conversations on the treatment and management of their sibling's RD. As discussed previously, these siblings are involved in caregiving and treatment practices at home, despite knowing little about the disease.

### 3.3 Negotiating Relationships Shaped by an RD

Siblings reflected on different relationships that aided in navigating their experience of having a brother or sister with an RD including: (1) relationship with parents and extended family; (2) friendships and concealment; (3) loving, protecting, and advocating for a sibling with an RD; and (4) barriers to a typical sibling relationship.

#### 3.3.1 Relationship With Parents and Extended Family

Although reflecting on how an RD diagnosis had affected their relationship with their parents and family, participants shared how they received less attention:

Lots of people now, including the family, their attention is more on him. They were always around me but now their attention isn't on me anymore… Everyone wants to know how it's going with him, what is he doing… The first while I was a little jealous because I was wondering why they don't like me anymore, but now I understand… I think they probably feel sorry for him and that's why they ask so much… Jane, 19F

Yoh, a lot has changed… I used to get everything I wanted when I asked for it. But now he takes up a lot of their time. Jake, 14M

This differential treatment sometimes placed strain on the relationship that participants had with their parents. Some siblings, like Jane, preferred to turn to other family members for comfort and reassurance:

I speak to my older sister about everything and then I usually cry… she understands me better than my mom would. Jane, 19F

Due to the nature of RDs, parents frequently accompany the sibling with an RD to the hospital for treatment. In this case, participants drew attention to the importance of support from extended family.

She [grandmother] is sometimes there when my parents are not. If they need to go somewhere and can't take us with, my grandmother says just leave them with me. Ella, 12F

She [sibling with rare disease] is with my grandma during the day while I am at school, so my granny helps a lot with that stuff. Layla, 18F

One participant noted how important the support of extended family would be in the future, when she would potentially assume the role of primary caregiver:

Then I decided the day that my mom isn't there anymore I can go to my aunts, and I can go to my grandmother. I can go to my mom's people, but maybe I should just stay right here where I am, and I must be strong. Ella, 12F

#### 3.3.2 Friendships and Concealment

In this study, most participants revealed that their friends were unaware of their sibling's diagnosis and that they had not disclosed to anybody outside their family. Participants were worried about their friends' reactions when learning about their sibling's diagnosis. Others feared their friends would disclose the information they shared with them:

No, they have never met him. Nobody knows that he is sick… Just the family in the house knows that he is sick… They [friends] are just going to laugh; they don't understand those kinds of things yet… I just know they won't understand. Max, 12M

I think it's better to keep it with me and my family. I think it's better that way because this time lately you can't trust a person. He or she will go post on social media and talk about it behind my back. Liam, 13M

When one participant wanted to speak to peers about his feelings, this fear led him to conceal the identity of his sibling who had been diagnosed with an RD:

I usually speak to my, there's a friend of mine next door… I didn't tell him that it is my little brother that has the disease… Yes, I don't usually tell him that it's my brother because I think he will not act the same way he was acting before when he heard that he has some disease that he was born with. Ethan, 12M

In these situations, where friends were unaware of the RD, participants tended to isolate themselves socially:

I am not really outside very much, the only time I am out I go to my cousin… I am more with him [brother with a rare disease] than I am with my other friends. Ella, 12F

#### 3.3.3 Loving, Protecting and Advocating for a Sibling With an RD

Participants described a close bond with their siblings with an RD:

Me and him, we get along very well. I understand him. If someone says something to him then I will be the one to help him to calm down. Most of the time he will call for me because he knows I understand him, and he understands me… I wouldn't want anyone else as a brother. Jane, 19F

I don't find him different from us. He's like he doesn't have any disease… I wish people know that if you have a brother or you have a sister with a disease or some sort of disease, don't treat them another way. They are a person like you, and you don't have to treat him the other way, because at the end of the day you are siblings. Ethan, 12M

During the interviews, participants were asked what advice they would give to somebody who also had a brother or sister with an RD. The excerpts below highlight the suggestions that siblings offered, and illustrate how the participants relate to their siblings with empathy and compassion:

Just be more patient. Spend lots of time with them and talk to them about their disease. Do anything you can to understand the disease better. Treat them right, they are only human. It can happen to any of us. Just try to understand and be there for them in the situation. Jane, 19F

Just be patient and try to be calm or reassuring to them that nothing is really wrong with them. That's all. Layla, 18F

Siblings tended to act as a defender for their brother or sister who had been diagnosed with an RD. Although reflecting on the attitudes of community members, participants noted that people would often stare in public, making them feel protective:

Most definitely when she was younger, before the operation. I know that people looked at us. I was just like, why, there is nothing to look at really. I think it was more angry for her, not angry at her, because I know she is a baby and she wouldn't understand why people would look at her differently. I know why they are looking and it's like, you don't know what's going on. You don't have to be judgmental. Layla 18F

Participants also reported that their brother or sister with an RD had been targeted by bullies, either at school or in their communities. In these instances, siblings described stepping in to defend and advocate for them and shared a need to raise awareness about RDs:

Other people bully and tease him because he is slow. We have been to the school over it because one group threatened him… For a long time, he was bullied at school, but he didn't want to tell us because they convinced him that his family can't do anything. One day he came home, and he was crying so my dad and I went to their house … we've had the police go there as well. Jane, 19F

#### 3.3.4 Barriers to a Typical Sibling Relationship

Siblings were originally guarded and uncomfortable sharing the challenges that they faced. They adopted a protective stance towards their diagnosed siblings and seemed to feel they were betraying them by speaking about their trials. Various aspects of an RD diagnosis made the sibling relationship difficult for participants. It was important to distinguish between challenges that were typical in a sibling relationship, such as arguing over TV shows, and those that were directly related to RD symptoms such as speech delays, behavioral problems, and restrictions of mobility:

It's not nice having a brother who can't speak and who you can't understand, Max. 12M

One foot is still a little bit sensitive. She doesn't want to stand so long. Sometimes I say, can we go walk a little bit, and she say no, it's still sore… Unless I push her in the wheelchair then we can go. Grant, 11M

We've noticed he has a very short temper, and he has started hitting … before he was on the pill they gave him now, he was very short. We couldn't say anything then he lashes out or goes off with his behavior. Jane, 19F

### 3.4 Managing the Emotional Impact of an RD

Siblings have a need for support from people who could understand their complex situations. This theme is illustrated by the following subthemes: (1) emotional complexity, (2) coping strategies; and (3) need for peer support.

#### 3.4.1 Emotional Complexity

Although describing their experience, participants often highlighted their emotions at the time of the interview, both positive and negative. Female participants spoke more openly about their emotional responses than male participants, who mostly responded with silence, fidgeting, and shrugging when asked about emotional experiences.

Siblings described feelings of sadness, worry, and concern when reflecting on medical issues:

I felt heartbroken. I was just sad that he wasn't like other babies… The fact that he couldn't sit up straight. There was one day where my mother came home from the hospital, she told me that his spine grew skew, and that's what caught me. Jake, 14M

My mom told me that I mustn't worry so much about my brother. I told her: ‘How can I not be worried about him?’ I am scared he is going to hurt himself when I am not there and then I won't even know anything about it. Ella, 12F

Siblings furthermore described feelings of anger that their daily routine was disrupted by behavioral difficulties or caregiving needs:

He just makes me really angry sometimes and then I want to shout at him. He yells at me and says stuff, because I don't understand him… The words that come out of his mouth, that where he says something and then a person can't understand him, that's what makes me angry. Max, 12M

Sometimes the I get angry because I don't have time for them. Ella, 12F

Despite the negative emotions discussed above, siblings also reflected positively on their experiences. Participants earnestly highlighted achievements, talents and positive personality traits of their brother or sister, speaking with pride:

He can write and build blocks. He is very smart with a lot of things … every day he comes with something new. A new word, a new thing. Max, 12M

She likes doing stuff with character and she learns stuff very quickly. It doesn't look like she even has a disability, because she can do everything except walking. Grant, 11M

Furthermore, siblings were hopeful and optimistic about the future of their brother or sister with an RD:

I'd say you mustn't worry about it because there will come someday where she will be able to walk or do anything else. Grant, 11M

#### 3.4.2 Coping Strategies

Siblings made use of various active and passive strategies to cope with their experiences. Jake, 14 M, highlighted religion as an important coping mechanism that aided him in his journey:

I will usually leave that for Sunday when we go to church and pray. We pray for him … just pray, pray pray.

Ethan, 12 M, on the other hand, preferred to participate in physical activities to “clear my mind”:

When I feel worried, I will maybe go to play sport to make me feel better or to jog… I think I am not a guy who talks too much. I am not really open to a person.

A common coping mechanism amongst siblings in this study was the tendency to internalize their emotions while isolating themselves.

I would just really block it out of my mind first. Then I think about it again, think when I am a bit calmer again. Layla, 18F

I don't like talking about things with my mouth. I don't really like speaking about these things. Ella, 12F

#### 3.4.3 Need for Peer Support

None of the participants in this study were part of a support group for siblings of an individual with an RD. When prompted about whether they would benefit from meeting other siblings of an individual with an RD, siblings spoke in favor of such initiatives:

I would really like to, yes. Then we can share our experiences with each other and then I can also learn something from them… if somebody that is very close to them also has a problem, then you understand how things change. Jane, 19F

As noted previously, participants also did not benefit from support from their friends, as they had not disclosed their sibling's RD diagnosis.

## 4 Discussion

This study's findings highlight key aspects of the experience of having a sibling with an RD in Cape Town, South Africa. What follows is a discussion of the findings in relation to the study's objectives: (a) exploring the challenges posed by having a sibling diagnosed with an RD; (b) understanding how siblings navigate challenges associated with having a brother or sister with an RD; and (c) investigating how family organization, such as communication processes, influences the experience of siblings.

### 4.1 The Challenges Posed by Having a Sibling With a RD

The extent to which siblings in this study assisted their parents, by acting as adjunct caregivers, corresponds to previous literature which indicated that siblings play a significant role in caretaking (Grant et al. 2021; Haukeland et al. 2021; Heaton et al. 2023; Hutson and Alter 2007; Malcolm et al. 2014). According to the Revised Theory of Creating a Tenuous Balance, siblings tend to take on responsibilities that were previously carried out by their parents and are expected to take on active caregiving roles for their brother or sister with an RD (Havill et al. 2019). Siblings become completely immersed in the role of caregiver, assume a pseudo parent‐like role, and can lose a sense of themselves as separate from the disease (Havill et al. 2019). Beyond the practical demands of caregiving, the findings suggest that siblings might perceive themselves as responsible for decisions on aspects of care that would ordinarily fall within the parental role. These experiences potentially led to tensions in parent‐sibling relationships, as noted in the findings. In this study, the tension described appeared to stem from differing views between sibling and parent on priorities in care, rather than from the burden of the caregiving tasks themselves. This suggests that parentification may involve not only instrumental tasks but also a sense of responsibility for the quality of care a brother or sister receives. Further research is needed to establish how commonly such tensions occur, and whether they also reflect broader difficulties in holding both sibling and parent‐like roles.

The concern that siblings do not have enough time to meaningfully participate in schoolwork, sport and extramural activities has previously been highlighted, as some siblings fail to attend school to take care of their brother or sister with an RD and neglect their own hobbies or interests (Grant et al. 2021; Haukeland et al. 2015; Heaton et al. 2023). In parallel, siblings in this study felt that they could only turn their attention to social and leisure activities once their brother or sister with an RD had been fully taken care of. This is a worrying finding, as several studies have illustrated the importance of free time and leisure activities in the emotional and cognitive development of adolescents (Ginsburg et al. 2007; McHale et al. 2001). Taking these findings and the experiences of siblings into consideration, it is of utmost importance for siblings to receive support in balancing caregiving duties and personal time to aid in their well‐being and development.

Throughout the accounts of siblings in this study, the lack of information and resources available on RDs in South Africa was evident. Participants in this study notably misunderstood the RD of their brother or sister and displayed immense uncertainty about diagnostic aspects such as symptom profiles, prognosis, and treatments. These findings echo that of Vatne et al. (2015) who found that siblings aged six years to 17 years misunderstood abstract medical concepts and generalized lay health beliefs to the complex condition of their brother or sister, regardless of age. The authors noted that the abstract nature of RD concepts could result in misunderstandings that persist into late adolescence (Vatne et al. 2015). However, it is important to note that siblings' understanding is shaped by the information available to them and the capacity of adults around them to communicate it effectively. As in the case of our study, where parents themselves had unanswered questions and limited knowledge of rare diseases, siblings may have few opportunities to obtain the clarity they seek. Educational initiatives provided by rare disease professionals and specialists, such as those offered by Rovira‐Moreno et al. (2021), could be beneficial in improving knowledge and management of psychosocial aspects of rare diseases. These programs could potentially be adapted to adolescents and young adults to alleviate uncertainties and cater to the informational needs of siblings.

Parent–child relationships and family bonds have been found to become markedly strained due to the demands of an RD (Atkins and Padgett 2024; Doyle and Werner‐Lin 2016). Although siblings in this study noted feelings of jealousy towards their brother or sister because of differential treatment, they understood the need for increased care and attention. These reflections align with observations from Hutson and Alter (2007), clarifying that the jealousy experienced by siblings was temporary and episodic. The relationship that siblings in the current study had with their friends was a complicated aspect of their experience. The reasons for hiding their sibling's diagnosis from their peers included a fear that their diagnosed sibling would be stigmatized or bullied. Contrasting with previous work, where siblings reported prejudice and discrimination after revealing their brother or sister's RD to peers and community members (Malcolm et al. 2014), siblings in this study chose not to disclose the diagnosis. This possibly novel experience of our sample resulted in siblings being cut off from the potential emotional support of their peer network.

The Revised Theory of Creating a Tenuous Balance notes that siblings experience strong feelings that include an array of negative emotions, such as anger, sadness, and fear (Havill et al. 2019). Since siblings are so intimately aware of the disease progression of their brother or sister with a rare disease, they experience considerable concern and worry (Grant et al. 2021; Haukeland et al. 2015; Hutson and Alter 2007; Malcolm et al. 2014). Siblings in this study voiced concerns at the prospect of their brother or sister needing to undergo painful treatments and the possible deterioration of their condition. Furthermore, Heaton et al. (2023) found that the possibility of stepping into the role of a fulltime caregiver results in the aggravation of worries and concerns from siblings. It is, therefore, to be expected that participants in this study were troubled by uncertainties regarding the support needs of their brother or sister with a rare disease and how this would be met in the future.

The Revised Theory of Creating a Tenuous Balance notes that siblings experience positive feelings of pride and protection over their brother or sister with a disease, in addition to the negative emotions described above (Havill et al. 2019). Grant et al. (2021) investigated the experiences and support needs of siblings who have a brother or sister with mucopolysaccharidosis and found that 90% of participants liked and felt proud of their diagnosed sibling. Likewise, siblings in this study earnestly highlighted the talents, achievements and personality traits of their diagnosed brother or sister. Participants furthermore noted that their brother or sister with a rare disease had taught them to be patient and empathetic towards others. These findings parallel those of Grant et al. (2021), in which 22% of adolescent and adult siblings felt that their diagnosed sibling had taught them valuable life lessons, such as being compassionate and helping those in need.

### 4.2 Navigating the Challenges of Having a Sibling With an RD

Siblings in this study used a selection of coping strategies to manage their experiences and, more specifically, the challenges associated with having a brother or sister with an RD. Participants clearly stated that they preferred using passive coping strategies, such as going for a walk, playing outside, or spending time alone. These findings align with those of Haukeland et al. (2015) who noted that it was more common for siblings to hide their emotion by repressing or ignoring unpleasant feelings than it was to share them with others.

The relationship that siblings have with extended family was discussed separately from those they share with their parents and friends. In this study, siblings turned primarily to grandparents, aunts, and uncles for instrumental and emotional support and not their parents or friends. This finding supports the notion expressed in the Revised Theory of Creating a Tenuous Balance that siblings rely on practical support from their family members while navigating the challenges associated with having a brother or sister with an RD (Havill et al. 2019). This finding shows that extended family members could serve as a potential domain for the strengthening of support networks for siblings.

It is crucial that siblings receive adequate psychosocial support to manage their stressors. Siblings have previously vocalized their feelings of otherness, stating that they had unique experiences that their loved ones were unable to understand (Grant et al. 2021; Heaton et al. 2023). This finding contradicts that of the Revised Theory of Creating a Tenuous Balance, which proposes that siblings turn to family, friends, and healthcare providers for social support (Havill et al. 2019). None of the siblings in this study attended any type of support group but reported that they would be interested in participating in one. In South Africa, there are numerous support groups that target individuals with RDs, and these are thoroughly indexed on the Rare Disease South Africa webpage which is open to access by the public. However, at the time of writing this article, none of these support groups catered for siblings.

### 4.3 Family Organization and the Sibling Experience

Parent–child communication in RD families has been a popular topic of investigation, and Haukeland et al. (2021) found that RD siblings scored significantly lower on the Parent–Child Communication Scale when compared to a control group from Norwegian elementary schools. Similarly, siblings in this study reported that their families rarely spoke about the RD of their brother or sister and that they did not feel comfortable enough to broach the subject with their parents. This lack of communication could contribute to further misconceptions and uncertainties about RDs as there are few opportunities for clarification.

To fully conceptualize the experience of having a sibling with an RD, it is important to consider the individual relationship that participants had with their diagnosed brother or sister. According to the Revised Theory of Creating a Tenuous Balance, siblings become more empathetic and mature, are committed to the wellbeing of their brother or sister, and advocate for them in public (Havill et al. 2019). In parallel, siblings in this study spoke highly of their brother or sister with an RD but also noted significant barriers within their sibling relationship. These barriers, which manifested in typical sibling activities, included mobility difficulties, speech impediments, and neurodevelopmental deficits. A growing body of research indicates the significance of sibling relationships in beneficial emotional and social development (Barberis et al. 2022; Yucel and Yuan 2015), and the limitations placed on RD sibling relationships thus raise concerns about the impact that these restrictions could have on sibling developmental outcomes.

## 5 Strengths and Limitations

This study is the first to investigate the experience of having a brother or sister with an RD in South African. Therefore, this study provides valuable insights into these challenges, experiences, and needs of siblings. The results suggest that siblings of an individual with an RD might need intervention, and this study can serve as a foundation for future research which aims to develop support programmes for this population group.

The sample of this study was geographically limited to Cape Town in the Western Cape, South Africa. Recruitment through Tygerberg Hospital may have resulted in a sample comprising individuals who were more engaged with healthcare services than the broader target population. According to the Health Professions Council of South Africa (2023), 37 genetic counselors were actively registered in the 2024/2025 reporting period, while Gomes et al. (2024) reported 17 active medical geneticist registrations in 2024. These figures suggest a limited specialist workforce relative to the needs of the approximately 4.2 million South Africans living with rare diseases (Malherbe 2023), although registration numbers alone do not indicate the geographical distribution of needs. Consequently, the experiences reported may differ from those of individuals with limited healthcare access. Although this study was exploratory in nature, future studies on siblings of an individual with an RD should aim to recruit participants across the nine provinces of South Africa and, where feasible, include individuals with varying levels of access to healthcare services to capture a broader account of sibling experiences.

Although participants provided valuable information on their experience of having a sibling with an RD, their accounts on emotional experiences were limited. This was especially true for male adolescent participants who were reluctant to reflect on their emotions and feelings. Future research could include narrative interviews to allow participants to describe the emotional significance of their experiences rather than recounting events. Additionally, repeat interviews could foster greater rapport between researchers and participants, enabling richer emotional exploration that may not emerge during a single interview.

## 6 Recommendations for Practice

The findings suggest that healthcare professionals such as genetic counselors should extend their practice beyond the traditional focus on the affected individuals and parents to explicitly engage siblings as members of the caregiving unit. Where appropriate, siblings' informational, psychosocial, and support needs should be assessed to allow for referral to age‐appropriate peer support services. In addition, efforts should be made to support open family communication and relational dynamics in RD families with a focus on the importance of age‐appropriate caregiving duties and reciprocal communication. To address the uncertainties and misunderstandings of siblings in this study, it would be crucial to equip siblings with the necessary health literacy to facilitate their coping. Educational programmes should be established in primary and/or secondary healthcare settings, such as those described by Rovira‐Moreno et al. (2021), to empower siblings and families through knowledge acquisition. These educational programmes could be supplemented by information on coping strategies and relevant RD organizations in South Africa.

## 7 Conclusion

This study is the first to investigate the experiences that accompany having a sibling with an RD in South Africa. The exploratory nature of this study allowed for in‐depth insights into the challenges, coping strategies, and family dynamics of these siblings. The findings assist in building a foundation for intervention development that targets siblings and RD families, which could lead to the much‐needed provision of support.

## Author Contributions

**Melissa Goosen:** conceptualization, investigation, writing – original draft, methodology, writing – review and editing, formal analysis, data curation, project administration, visualization. **Shahida Moosa:** supervision, conceptualization, writing – review and editing, methodology, resources. **Chrisma Pretorius:** supervision, writing – review and editing, conceptualization, methodology, validation, resources.

## Funding

The authors have nothing to report.

## Ethics Statement

Ethical approval to conduct the study was received from Stellenbosch University's Health Research Ethics Committee (ethics reference number S24/01/015) as well as the Western Cape Health Department and Tygerberg Hospital. All participants provided informed consent to participate. The identities of participants are anonymous, and pseudonyms have been used to present the study results. All identifying information has been removed from quotations.

## Consent

Consent for publication has been collected from all participants.

## Conflicts of Interest

The authors declare no conflicts of interest.

## Supporting information


**Data S1:** Semi‐Structured Interview Schedule: Ages 10–13 Years.

## Data Availability

The data that support the findings of this study are available on request from the corresponding author. The data are not publicly available due to privacy or ethical restrictions.
