# Supplementary material for: Growing up in a Rare Disease Family: The Perspectives of Adolescent and Young Adult Siblings
Source: J Genet Couns. 2026 Nov 13;35(6):e70328. doi: 10.1002/jgc4.70328 (PMC13768569; doi:10.1002/jgc4.70328)
Supplement: Supplementary file 1 [file JGC4-35-0-s001.docx]

**Semi-Structured Interview Schedule: Ages 10–13 Years**

**A. Family Structure**

“I’m going to draw a picture that we can use to talk about your family.”

Following the interview technique used by Heaton et al. (2023), the interviewer will draw a family tree as the participant describes their family structure. This family tree will be used to guide the interview and to aid in contextualising family processes during data analysis.

1. Who do you live with?
2. How many siblings do you have?
3. Who is the oldest sibling? How old are your other brothers and sisters? (if applicable)
4. Who in your family has a rare disease?

Reminder: A rare disease is a disease that very few people in the world have. Less than in every 2000 people have a rare disease. Examples include Ehlers-Danlos syndrome, Duchenne muscular dystrophy and Fanconi anaemia.

5. How do you get along with [sibling’s name]?

**B. General Experience**

6. Tell me more about what it’s like having a brother or sister with a rare disease?

What do you like about it?

What do you not like about it?

What is difficult about having a brother or sister with this disease?

7. Has anything changed since [sibling’s name] was diagnosed? How have things changed?
8. How do your parents treat you compared to [sibling’s name]?
9. How do your friends behave in front of [sibling’s name]? In other words, how do they treat them?

**C. Responsibilities**

10. Who takes care of [sibling’s name]?
11. Do you help take care of [sibling’s name]?

If yes:

12. What do you have to do when you take care of [sibling’s name]?
13. Tell me more about what it’s like taking care of [sibling’s name]?

What do you like about taking care of them?

What is difficult about taking care of them?

14. How often do you take care of them?
15. How does taking care of your brother or sister change your day? Do you have time for school work or to see your friends?

**D. Communication Processes**

16. Can you remember when you first learned that [sibling’s name] had this disease?

Prompts:

Who told you about it?

How old where you when you were told about it?

17. What did you think or feel when you heard about the disease the first time?
18. How often does your family talk about [sibling’s name]’s disease?

If not often, why do you think this is?

Have you tried to find out more about [sibling’s name]’s disease yourself?

Who do you talk to when you have questions or feel worried about this disease?

**E. Final Thoughts**

19. What do you wish other people knew about what it’s like to have a brother or sister with a rare disease?
20. How do you think other people can help you to make growing up with a brother or sister with a rare disease easier?
21. What would you say to help other children who have a brother or sister with a rare disease?

**Semi-Structured Interview Schedule: Ages 14–24 Years**

**A. Family Structure**

“I’m going to draw a picture that we can use to talk about your family.”

Following the interview technique used by Heaton et al. (2023), the interviewer will draw a family tree as the participant describes their family structure. This family tree will be used to guide the interview and to aid in contextualising family processes during data analysis.

1. Who do you live with?
2. How many siblings do you have?
3. Who is the oldest sibling? How old are your other siblings (if applicable)
4. Who in your family has a rare disease?

Reminder: A rare disease is a disease that affects less than 1 in every 2000 people. Examples include Ehlers-Danlos syndrome, Duchenne muscular dystrophy and Fanconi anaemia.

5. What is your relationship like with your sibling who has a rare disease?

**B. General Experience**

6. Tell me about your experience of having a sibling with a rare disease?

Prompts:

What do you like about having a sibling with this disease?

What do you not like about having a sibling with a rare disease?

What do you find difficult about having a sibling with this disease?

7. Tell me more about your sibling with a rare disease?

Prompts:

What do they struggle with?

What do they need help with?

What, if anything, has changed since [sibling’s name] was diagnosed?

8. How do your parents treat you in comparison to [sibling’s name]?
9. How do your friends and members of your community treat your sibling with a rare disease?

**C. Responsibilities**

10. Who takes care of your sibling with a rare disease?
11. Do you help take care of your sibling?

If yes:

12. What responsibilities do you have when you take care of your sibling?
13. Tell me more about your experience of taking care of your sibling?

Prompts:

What do you like about taking care of [sibling’s name]?

What do you find difficult about taking care of [sibling’s name]?

14. How often do you take care of them?
15. Does taking care of your sibling affect your daily routine in terms of school work and socialising? How?

**D. Communication Processes**

16. Can you remember when you first learned that [sibling’s name] had this disease? Who Prompts:

Who told you about it?

How old were you when you were told about it?

17. What did you think or feel when you heard this news the first time?
18. How often does your family talk about [sibling’s name]’s disease?

Prompt: If not often, why do you think this is?

19. Have you looked for information about [sibling’s name] yourself?
20. Who do you talk to if you have questions or feel worried about this disease?

**E. Final Thoughts**

21. What do you wish other people knew about your experience of having a sibling with a rare disease?
22. How do you think other people can help you to make your experience easier?
23. What advice would you give to other children/adolescents/young adults who have a sibling with a rare disease?
